# John Tilsley

**Published:** 1900-12

**Authors:** 


### JOHN TILSLEY.

Mr. John Tilsley died at the residence of his father, George Street, Bath, on October 28th, from dysentery, in his thirty-second year. The deceased received his medical education at University College, Bristol, and took several prizes and scholarships. He qualified L.R.C.P. Lond. and M.R.C.S. Eng. in 1894, and after holding resident appointments at the Bristol General Hospital, proceeded as a medical missionary to Kiu-Kiang, China. On the outbreak of the recent disturbances there he was warned to leave by the British Consul, and consequently with his wife and child he started for England. After recovering from an attack of fever contracted at Shanghai, they proceeded on their journey. At Colombo, however, Mr. Tilsley unfortunately fell a victim to dysentery; but rallying, he continued the voyage, only to die the day after arriving at Southampton.

